# Vitamin D Deficiency and Its Impact on Prediction and Treatment of Postoperative Hypocalcemia in a Cohort of Patients Undergoing Total Thyroidectomy

**DOI:** 10.7759/cureus.80220

**Published:** 2025-03-07

**Authors:** Vijai Radhika, Anoop Pillai, Ekalavyan Jayaraj, Riju Ramachandran, Renjitha Bhaskaran

**Affiliations:** 1 General Surgery, Amrita Institute of Medical Sciences, Kochi, IND; 2 General Surgery, Amrita Institute of Medical Sciences and Hospital, Kochi, IND; 3 Biostatistics, Amrita Institute of Medical Sciences and Research Center, Kochi, IND

**Keywords:** calcium homeostasis, postoperative hypocalcemia, supplementation, total thyroidectomy, vitamin d deficiency

## Abstract

Background: Postoperative hypocalcemia is a common complication following total thyroidectomy, leading to extended hospital stays, decreased patient quality of life, and increased healthcare costs. Recent research has explored the role of preoperative vitamin D deficiency in exacerbating this risk due to its impact on calcium homeostasis.

Methods: A prospective study was conducted at a tertiary care hospital in Southern India from March 2023 to May 2024. Patients undergoing total thyroidectomy were categorized into three groups based on their preoperative vitamin D status: Group A (Vitamin D sufficient), Group B (Vitamin D deficient with preoperative supplementation), and Group C (Vitamin D deficient without supplementation). Serum calcium levels were monitored preoperatively and postoperatively to assess for hypocalcemia.

Results: A total of 84 patients were included: Group A (n=38), Group B (n=23), and Group C (n=23). Preoperative vitamin D levels were significantly lower in Groups B and C compared to Group A. The incidence of postoperative hypocalcemia was highest in Group C (n=23; 56.5%), followed by Group A (n=38; 42.1%) and Group B (n=23; 34.8%), although differences were not statistically significant (p=0.315). However, clinical hypocalcemia was significantly higher in Group C (n=23; 56.5%) compared to Group A (n=38; 23.7%) and Group B (n=23; 21.7%) (p=0.013).

Conclusion: Preoperative vitamin D deficiency is associated with an increased risk of postoperative hypocalcemia following total thyroidectomy. Preoperative supplementation in vitamin D-deficient patients may mitigate this risk, suggesting a potential benefit in routine assessment and correction of vitamin D status before surgery to optimize patient outcomes.

## Introduction

Total thyroidectomy is the surgery commonly performed in most surgical centers for the treatment of thyroid cancer, Graves’ disease, and large goiters. Complications such as hematoma with airway compression, hoarseness of voice, and ventilation problems due to recurrent laryngeal nerve (RLN) injury and hypocalcemia rarely occur, causing morbidity.

Postoperative hypocalcemia is a common complication of total thyroidectomy, with reported incidence rates up to 60% [1]. It can lead to prolonged hospital stay, decreased quality of life for patients, and increased healthcare costs. While the etiology of post-thyroidectomy hypocalcemia is multifactorial, recent research has focused on the potential role of preoperative vitamin D status [2].

Hypocalcemia can be either permanent or temporary and is caused by the inadvertent removal or ischemia of the parathyroid glands. In some patients, there is a transient period of hypocalcemia, despite preserving the parathyroid glands and their vascularity. Hypocalcemia is often asymptomatic or causes mild symptoms such as perioral or distal acral paresthesia. Painful cramps, tetany, and convulsion occur when serum calcium levels are less than 7.6 gm/dl and can limit the daily activities of such patients [3].

Vitamin D plays a crucial role in calcium homeostasis by enhancing intestinal calcium absorption and regulating parathyroid hormone (PTH) secretion. Its active form is synthesized in the kidney by a PTH-dependent process, which is responsible for the increase in gastrointestinal absorption of calcium and renal resorption of calcium and phosphate [4]. Because of these actions, the preoperative vitamin D level may have a profound impact on the perioperative kinetics of calcium and PTH after total thyroidectomy [5]. In regions with sufficient vitamin D, a normal level for an average individual is considered to be 30 ng/mL [6]. However, in countries where the population is deficient in vitamin D, a serum level of less than 20 ng/mL is generally accepted as a deficiency of vitamin D [7].

In vitamin D deficiency (VDD), compensatory mechanisms, including secondary hyperparathyroidism, maintain normal calcium levels. However, these mechanisms may be disrupted following thyroidectomy, potentially increasing the risk of postoperative hypocalcemia.

Several studies have examined the relationship between preoperative vitamin D levels and postoperative hypocalcemia, yielding mixed results. Some researchers have found a significant association between low vitamin D levels and an increased risk of hypocalcemia, while others report no correlation.

For example, Kirkby-Bott et al. observed that patients with vitamin D levels greater than 50 nmol/L experienced a lower incidence of postoperative hypocalcemia [5]. In a meta-analysis involving 3,037 total thyroidectomy cases, Sanabrai et al. found that consistent supplementation with calcium and/or vitamin D resulted in a 25% reduction in the risk of postoperative hypocalcemia [8]. In contrast, Erbil et al. found that patients with vitamin D levels below 15 ng/mL were significantly more likely to develop hypocalcemia after thyroidectomy [9].

In a retrospective study involving 150 patients observed between 2007 and 2013, Cherian et al. found that VDD, defined as a vitamin D concentration below 20 ng/ml, did not increase the risk of postoperative hypocalcemia in individuals who underwent thyroidectomy. However, this study included all patients who underwent thyroidectomy [10]. These conflicting findings highlight the need for further investigation into this important clinical question.

This study aims to investigate the relationship between preoperative vitamin D status and the incidence of postoperative hypocalcemia in patients undergoing total thyroidectomy. Additionally, we aim to evaluate the effectiveness of preoperative vitamin D supplementation in reducing this risk. By addressing these questions, we hope to contribute to the development of evidence-based strategies for preventing postoperative hypocalcemia and improving patient outcomes following thyroid surgery.

## Materials and methods

Study design

A prospective study was conducted to evaluate the effects of vitamin D on patients undergoing total thyroidectomy at the Department of General Surgery. The study took place from March 2023 to May 2024 at a tertiary care hospital in Southern India (Clinical Trials Registry number: CTRI/2023/04/051296).

Study population

The study included all patients scheduled for total thyroidectomy for benign thyroid disorders, excluding those who had undergone previous thyroid surgery, had a history of irradiation, were under 12 years of age, had central or lateral neck dissection, had coexisting parathyroid disease, or were taking medications affecting calcium homeostasis such as calcium or vitamin D supplements, lithium, bisphosphonates, or diuretics.

The study participants were categorized into two main groups based on their vitamin D levels: vitamin D sufficient (Group A), defined as having a 25-hydroxyvitamin D level greater than 20 ng/mL, and vitamin D deficient (less than or equal to 20 ng/mL). The vitamin D-deficient patients were further divided into two subgroups using lots: one group received preoperative vitamin D supplementation (Group B) and the other did not (Group C). Group B patients were administered at least four doses of vitamin D (cholecalciferol 60,000 IU once per week for four weeks) before surgery. Thus, the final grouping for the study included Group A (vitamin D sufficient), Group B (vitamin D deficient with preoperative vitamin D supplementation), and Group C (vitamin D deficient without preoperative supplementation). A pilot study with 10 patients per group was conducted, which helped determine a sample size of 69.

Preoperative serum corrected calcium and vitamin D levels were measured, and routine preoperative workup was done for assessing anesthetic fitness for surgery. The patients then underwent total thyroidectomy. All the thyroidectomies were performed by the senior experienced surgeons in the department. In all the patients, the parathyroid glands, including their vasculature, were clearly identified and preserved. Postoperatively, the patients were monitored for clinical and biochemical hypocalcemia on postoperative days (PODs) 1, 2, and 3. The normal range of serum corrected calcium is 8.6-10.2 mg/dL.

Clinical hypocalcemia was characterized by a decrease in serum corrected calcium levels to below 8.6 mg/dL, accompanied by symptoms such as mild perioral and peripheral numbness, a positive Chvostek’s sign, or even severe tetany. On the other hand, biochemical hypocalcemia was characterized by a reduction in serum corrected calcium levels to below 8.6 mg/dL without the presence of any hypocalcemic symptoms.

Serum corrected calcium was measured every morning until discharge. Patients were discharged after 48-72 hours if corrected calcium was within normal limits. Patients who developed hypocalcemia were treated with oral calcium supplementation (each film-coated tablet containing 1250 mg of calcium carbonate from an organic source, powdered oyster shell, equivalent to 500 mg of elemental calcium, along with 250 IU of vitamin D3 IP; dosed two tablets, two times a day) or intravenous calcium (10 mL of 10% calcium gluconate injection, administered over 10-20 minutes) as needed. Patients in Group B were continued on vitamin D for two more weeks, while those in Group C were supplemented with vitamin D (cholecalciferol 60,000 IU once a week for six weeks).

Study measures

Data about age, sex, pre- and postoperative calcium levels, vitamin D levels, and hypocalcemia (both clinical and biochemical) were recorded on an Excel sheet. Patients were discharged with serum corrected calcium levels of 8.6 mg/dL or more.

Sample size

Before initiating the dissertation study, a pilot study was conducted to determine the sample size. In this pilot study, the incidence of postoperative hypocalcemia was found to be 52% in the vitamin D deficient (VDD) group who received supplementation, 89% in the preoperative VDD group who did not receive supplementation, and 10% in the vitamin D non-deficiency group. With a desired power of 80% and a 95% confidence level, the required sample sizes for each group were 18, 23, and 5, respectively. Based on the largest required sample size, the proposed total sample size was set at 69 patients, with a minimum of 23 patients per group.

Statistical analysis

Descriptive statistics were expressed as median (Q1-Q3) for continuous variables and as frequency and percentage for categorical variables. The Kruskal-Wallis test was used to compare continuous variables between the three groups. The chi-square test was used to compare categorical variables. The Friedman test was used to assess changes in calcium levels over time within each group. A p-value <0.05 was considered statistically significant. All analyses were performed using IBM SPSS Statistics for Windows, Version 20.0 (Released 2011; IBM Corp., Armonk, New York, United States).

## Results

A total of 84 patients were included in the study: Group A (n=38), Group B (n=23), and Group C (n=23). The demographic characteristics of the patients were similar across the three groups. Participants' ages ranged from 25 to 81 years, with a mean age of 57.74 years in Group A; from 27 to 67 years, with a mean age of 47.17 years in Group B; and from 19 to 63 years, with a mean age of 46.48 years in Group C (Table 1).

**Table 1:** Distribution of age in each group.

Group	Frequency	Mean age	SD
A	38	57.74	11.636
B	23	47.17	10.408
C	23	46.48	10.900

In Group A, the majority of participants were female, comprising 97.4% of the group, while a small proportion, 2.6%, were male. A similar trend was observed in Group B, where 95.7% of participants were female and 4.3% were male. However, in Group C, there was a notable difference in gender distribution, with 78.3% of participants being female and 21.7% male (Table 2). 

**Table 2:** Distribution of gender in each group.

Gender	Group		
	Group A	Group B	Group C
Female	37 (97.4%)	22 (95.7%)	18 (78.3%)
Male	1 (2.6%)	1 (4.3%)	5 (21.7%)

The median (Q1-Q3) preoperative vitamin D in Group A (n=38) was 24.7 (22.4-30.4), in Group B (n=23) was 13.9 (9.6-16.8), and in Group C (n=23) was 14.8 (10.4-17.4) (Table 3).

**Table 3:** The median (Q1-Q3) preoperative vitamin D levels.

Group	n	Median (Q1-Q3)
A	38	24.7 (22.4-30.4)
B	23	13.9 (9.6-16.8)
C	23	14.8 (10.4-17.4)

When comparing postoperative hypocalcemia between the groups, the proportion of patients who developed postoperative hypocalcemia was higher in Group C (n=23) (13; 56.5%), compared to Group A (n=38) (16; 42.1%) and Group B (n=23) (8; 34.8%). However, this difference did not reach statistical significance (p<0.315) (Table 4).

**Table 4:** Incidence of postoperative hypocalcemia.

Postoperative hypocalcemia	Group A, n (%)	Group B, n (%)	Group C, n (%)	p value
Yes	16 (42.1)	8 (34.8)	13 (56.5)	0.315
No	22 (57.9)	15 (65.2)	10 (43.5)	

There was a significantly increased number of patients in Group C (n=23) (14; 60.9%) requiring supplementation with calcium in the postoperative period as compared to Group A (n=38) (15; 39.5%) and Group B (n=23) (7; 30.4%) (p<0.097) (Table 5). 

**Table 5:** The proportion of patients requiring postoperative calcium supplementation.

Postoperative calcium supplements	Group A, n (%)	Group B, n (%)	Group C, n (%)	p value
Yes	15 (39.5)	7 (30.4)	14 (60.9)	0.097
No	23 (60.5)	16 (69.6)	9 (39.1)	

There was a significantly higher proportion of patients having clinical hypocalcemia in Group C (n=23) (13; 56.5%) compared to Group A (n=38) (9; 23.7%) or Group B (n=23) (5; 21.7%) (p<0.013) (Table 6).

**Table 6:** Comparison of clinical hypocalcemia between groups.

Clinical hypocalcemia	Group A, n (%)	Group B, n (%)	Group C, n (%)	p value
Yes	9 (23.7)	5 (21.7)	13 (56.5)	0.013
No	29 (76.3)	18 (78.3)	10 (43.5)	

Table 7 shows the median levels of serum calcium at different time points. Preoperatively, no significant difference was found among the groups (p<0.228). However, on POD 1, there was a significantly lower calcium level (p<0.005), with Group C (n=23) showing the lowest median levels (8.4 mg/dL, Q1-Q3: 8.0-8.8). On POD 2, there was no significant difference among the groups with p<0.092, and on POD 3, no significant difference was found (p<0.064), though Group C (n=23) had the lowest median levels. These findings indicate that the most critical period for the drop in calcium levels and the need for supplementation is immediately post-surgery, particularly in Group C (n=23) (Table 7).

**Table 7:** Median serum calcium levels (mg/dL) at different time points. POD: postoperative day.

Preoperative calcium	Group	n	Median (Q1-Q3)	Mean	p value
Preoperative	A	38	9.3 (9.0-9.6)	9.4	0.228
	B	23	9.1 (9.0-9.3)	9.2	
	C	23	9.3 (9.1-9.4)	9.2	
POD 1	A	38	8.8 (8.4-9.1)	8.8	0.005
	B	23	8.9 (8.6-9.0)	8.8	
	C	23	8.4 (8.0-8.8)	8.4	
POD 2	A	38	8.5 (8.2-9.3)	8.7	0.092
	B	23	8.7 (8.4-8.9)	8.7	
	C	23	8.4 (7.9-8.9)	8.4	
POD 3	A	38	8.8 (8.0-9.3)	8.7	0.064
	B	23	8.8 (8.4-9.1)	8.8	
	C	23	8.1 (7.6-8.9)	8.3	

## Discussion

This study demonstrates a significant association between preoperative VDD and the risk of postoperative hypocalcemia following total thyroidectomy. Patients with VDD who did not receive preoperative supplementation (Group C, n=23) had the highest incidence of postoperative hypocalcemia (56.5%) compared to those with normal vitamin D levels (Group A (n=38), 42.1%) and those who received preoperative supplementation (Group B (n=23), 34.8%).

A pilot study was conducted for the purpose of sample size estimation in a tertiary care hospital in South India during the period of April to May 2022. The pilot study showed an association between VDD and postoperative hypocalcemia, with a significant decrease in postoperative hypocalcemia favoring the group with preoperative vitamin D supplementation (p<0.003).

The protective effect of preoperative vitamin D supplementation is evident in the lower rates of hypocalcemia and calcium supplementation requirements in Group B (n=23) compared to Group C (n=23). This finding suggests that correcting VDD before surgery may help mitigate the risk of postoperative hypocalcemia. The mechanism underlying this protective effect likely involves the optimization of calcium homeostasis prior to the surgical insult.

Several studies have examined the role of preoperative vitamin D levels as a risk factor for hypocalcemia after total thyroidectomy, with mixed results. Lin et al. found no predictive value of vitamin D levels on postoperative hypocalcemia in a cohort where patients were routinely given calcium and vitamin D postoperatively [11]. Similarly, Chia et al. reported no association, though their study included a heterogeneous group of patients [12].

In some studies, VDD has shown predictive value for postoperative hypocalcemia. In a retrospective study covering 166 total thyroidectomies from 2006 to 2009, Kirkby-Bott et al. found that patients with VDD had a greater likelihood of developing hypocalcemia compared to those with sufficient vitamin D levels [5]. Similarly, Erbil et al. reported that patients with vitamin D levels below 15 ng/mL had a significantly higher rate of post-thyroidectomy hypocalcemia [9].

In a meta-analysis of 10 randomized controlled trials by Xing et al., total thyroidectomy patients provided with prophylactic calcium and vitamin D supplementation were at a decreased risk of transient postoperative hypocalcemia compared to those with calcium supplementation alone [13].

In another meta-analysis involving 3,037 total thyroidectomy cases, Sanabrai et al. found that regular supplementation with calcium and/or vitamin D led to a 25% decrease in the risk of postoperative hypocalcemia [8].

In a similar study, it was found that serum preoperative vitamin D levels were significantly lower in the group of patients who developed hypocalcemia (19.57 (7.10-24.84) ng/mL) compared to normocalcemic patients (28.7 (23.5-40.5) ng/mL) (p=0.012) [14].

The significantly lower calcium levels observed in Group C (n=23) on POD 1 further support the hypothesis that VDD increases susceptibility to acute changes in calcium homeostasis following thyroidectomy. This early drop in calcium levels may represent a critical period for intervention to prevent symptomatic hypocalcemia.

The mechanism by which VDD increases the risk of postoperative hypocalcemia likely involves the disruption of calcium homeostasis. In vitamin D-deficient states, secondary hyperparathyroidism develops to maintain normal calcium levels. Following thyroidectomy, the sudden loss of parathyroid function, even if temporary, may lead to a more pronounced drop in serum calcium levels in these patients. Preoperative vitamin D supplementation may help normalize this compensatory mechanism, thereby reducing the risk of postoperative hypocalcemia.

Our study has several strengths, including its prospective design, randomization of vitamin D-deficient patients to supplementation or no supplementation groups, and comprehensive follow-up of calcium levels. The inclusion of a control group with normal vitamin D levels provides a valuable reference for comparing outcomes.

However, there are also limitations to consider. The single-center design may limit the generalizability of our findings to other populations or healthcare settings. The relatively small sample size, while meeting our calculated requirements, may have limited our ability to detect smaller differences between groups. Additionally, the open-label nature of the intervention could have introduced some bias, although this was mitigated by blinding the laboratory personnel analyzing calcium levels.

## Conclusions

Preoperative VDD is associated with an increased risk of postoperative hypocalcemia in patients undergoing total thyroidectomy. Supplementing vitamin D in deficient patients before surgery may help reduce this risk. These findings suggest that routine preoperative vitamin D assessment and correction of deficiency should be considered as part of the management strategy for patients undergoing total thyroidectomy.

This study emphasizes the significant role of preoperative VDD in the development of postoperative hypocalcemia. Although the proportion of patients experiencing postoperative hypocalcemia was highest in the vitamin D-deficient group (Group C), this difference did not reach statistical significance when compared to the other groups (A and B). Nevertheless, a marked increase in the need for calcium supplementation was observed in Group C, highlighting the importance of addressing VDD before surgery to minimize postoperative complications.

The results suggest that correcting VDD may help stabilize calcium levels immediately after surgery, especially in the critical first 24 hours. While no significant differences in calcium levels were observed on POD 2 and POD 3, the early postoperative period remains a crucial time for intervention in vitamin D-deficient patients.

The findings of this study have important clinical implications for managing thyroidectomy patients. Identifying and correcting VDD preoperatively could reduce the incidence of postoperative hypocalcemia, potentially improving patient outcomes and reducing healthcare costs related to extended hospital stays and the treatment of symptomatic hypocalcemia.
